# Perceptions of healthcare professionals towards palliative care in internal medicine wards: a cross-sectional survey

**DOI:** 10.1186/s12904-021-00787-2

**Published:** 2021-06-30

**Authors:** Jason Tay, Scott Compton, Gillian Phua, Qingyuan Zhuang, Shirlyn Neo, Guozhang Lee, Limin Wijaya, Min Chiam, Natalie Woong, Lalit Krishna

**Affiliations:** 1 Department of Internal Medicine, Singapore General Hospital, Outram Rd, Singapore, 169608 Singapore; 2 Duke-NUS Medical School, 8 College Road, Singapore, 169857 Singapore; 3 Division of Supportive and Palliative Care, National Cancer Centre Singapore, 11 Hospital Crescent, Singapore, 169610 Singapore; 4 Department of Infectious Diseases, Singapore General Hospital, Outram Road, Singapore, 169608 Singapore; 5 Division of Cancer Education, National Cancer Centre Singapore, 11 Hospital Crescent, Singapore, 169610 Singapore; 6 Palliative Care Institute Liverpool, Academic Palliative & End of Life Care Centre, University of Liverpool, United Kingdom Cancer Research Centre, University of Liverpool, 200 London Rd, Liverpool, L3 9TA UK; 7 Centre for Biomedical Ethics, National University of Singapore, Blk MD 11, 10 Medical Drive #02-03, Singapore, 117597 Singapore; 8 PalC, The Palliative Care Centre for Excellence in Research and Education, 10 Jalan Tan Tock Seng, Singapore, 30843 Singapore

**Keywords:** Education and training, End of life, Internal medicine, Nurses, Palliative care, Palliative medicine, Physicians, Quality of life

## Abstract

**Background:**

The extension of palliative care services to meet the needs of patients with chronic non-malignant life-limiting conditions faces misconceptions amongst healthcare professionals. A study of prevailing perceptions of healthcare professionals on this wider palliative care service was thus conducted to identify current obstacles, guide the education of local healthcare professionals and improve service accessibility.

**Methods:**

A cross-sectional study was carried out at the Singapore General Hospital. An anonymised and close-ended online questionnaire was disseminated to 120 physicians and 500 nurses in the Department of Internal Medicine. The online survey tool focused on participant demographics; perceptions of palliative care and its perceived benefits; roles and indications; and attitudes and behaviours towards palliative care referrals.

**Results:**

Forty four physicians and 156 nurses suggested that care of terminally ill patients with chronic non-malignant life-limiting conditions are compromised by concerns over the role of palliative care in non-cancer care and lapses in their prognostication and communication skills. Respondents also raised concerns about their ability to confront sociocultural issues and introduce palliative care services to patients and their families.

**Conclusions:**

Gaps in understanding and the ability of nurses and physicians to communicate end of life issues, introduce palliative care services to patients and their families and confront sociocultural issues suggest the need for a longitudinal training program. With similar concerns likely prevalent in other clinical settings within this island nation, a concerted national education program targeting obstacles surrounding effective palliative care should be considered.

## Background

Whilst palliative care services have been credited with improving the quality of life of terminally ill cancer patients and providing holistic support for families, its role in the care of patients with non-cancer diagnoses and chronic life-limiting conditions remains poorly understood by healthcare professionals [1–5]. A study by the Lien Foundation in Singapore found that 74% of the 200 physicians and 46% of the 400 nurses surveyed reported inadequate understanding of palliative care’s role [5]. These gaps in understanding and misconceptions on the role of palliative care interventions have been identified as significant barriers to referrals and the utilisation of palliative care services [3, 4].

With data suggesting that unmet needs, symptom severity and psychosocial distress of patients with end-stage non-malignant diseases are comparable to patients with cancer, poor utilisation of palliative care and delayed referrals are likely to result in prolonged symptom burden. The need to improve awareness of palliative care’s role amongst healthcare professionals in internal medicine wards is evident [6–10]. This has come into sharp focus in Singapore as the Department of Internal Medicine at the Singapore General Hospital, a local tertiary hospital with an inpatient palliative care consultation service focused on non-cancer care, seeks to extend its palliative care services to chronic non-malignant life-limiting conditions.

Serving as primary users of this extended service, the team at the Department of Internal Medicine sought to survey and better understand perceptions, and misconceptions, of palliative care amongst physicians and nurses in the department, with careful attention towards conceptual differences raised between the two populations. Data accrued will be used to further guide and inform their palliative care education.

## Methods

To design the survey, the research team reviewed recent studies on perceptions of palliative care amongst healthcare professionals [11–26]. These reviews allowed the research team to draw up a set of survey questions focused on answering the primary research question “*how do physicians and nurses perceive palliative care and its role in the management of patients with terminal illnesses*?” The potential questions for the survey were discussed amongst the research team and adapted to account for practice and work schedules.

A cross-sectional study involving physicians (*n* = 120) and nurses (*n* = 500) from the Department of Internal Medicine was carried out at Singapore General Hospital. Study exemption was accorded by the SingHealth Centralised Institutional Review Board (CIRB reference no. 2019/2396).

All physicians – including consultants, registrars, medical and house medical officers, and nurses – including enrolled, registered nurses, nurse clinicians and managers were invited to participate in this anonymised and close-ended online questionnaire.

The survey was categorised into three distinct domains:

1. Participant demographics relating to clinical and palliative care experience.
2. Perceptions of palliative care with a focus on perceived benefits,^1^ roles and indications of consultation referrals.^2^
3. Self-reported behaviours so as to identify potential obstacles in initiating palliative care – these include participants’ confidence in explaining the role of palliative care to patients,^3^ confusion with palliative terminologies^4^ and views on palliative care training and utilisation.^5^

Email invitations replete with a participant information sheet and a link to the self-administered survey were disseminated by the department secretary and nursing managers. Two reminder emails were sent out over the course of the next 2 weeks.

Categorical variables (such as demographic data) and ordinal variables (such as Likert scale ratings) between physicians and nurses were analysed using chi-square or Wilcoxon Rank Sums tests. To aid in the interpretation of the ordinal responses, means and standard deviations are presented. All *p*-values are associated with non-parametric tests. All analyses were conducted using SPSS Software (SPSS, Inc., Chicago, IL, USA, version 26).

## Results

### Demographics, clinical and palliative care experience

Of the 200 healthcare professionals who participated in the study, 44 (22%) were physicians and 156 (88%) were nurses (Table 1). The response rate was 32.3%.

**Table 1:** Participant characteristics

	Physicians (***n*** = 44)	Nurses (***n*** = 156)	***p***-value
Years’ Experience* n(%) selecting			
< 5 Years	22 (48.9)	32 (20.5)	
> 5 to 10 Years	15 (33.3)	65 (41.7)	0.001
> 10 to 20 Years	5 (11.4)	50 (32.1)	
> 20 years	2 (4.4)	9 (5.8)	
At least “frequently” interacts with terminally ill patients* n(%) agree	35 (79.5)	111 (71.2)	0.268
Where did you first learn of palliative care?* n(%) selecting			
Medical or Nursing School	33 (76.7)	88 (58.3)	
Workplace	7 (16.3)	62 (41.1)	0.002
Family or friends	2 (4.7)	1 (0.7)	
Media	1 (2.3)	0 (0.0)	
Are you aware of any palliative care services in your hospital/institution?* n(%) yes			
When you hear of the term hospice, what do you first think of?* n(%) selecting	43 (97.7)	143 (94.7)	0.400
A place for cancer patients	0 (0.0)	14 (9.0)	
A place where all hope is lost	2 (4.5)	6 (3.8)	0.109
A place for pain medication titration and control	4 (9.1)	5 (3.2)	
A place for palliation and optimising quality of life	38 (86.4)	128 (82.1)	
A place for elderly patients	0 (0.0)	3 (1.9)	

† Based on the following options: “very frequent”; “frequent”; “occasionally”; “rarely”; “never”

All p-values for * obtained from chi square test

Nearly 50% of the physicians who responded had less than 5 years of clinical experience whilst approximately 80% of the nurses who responded had more than 5 years of clinical experience. Both groups reported having frequent interactions with terminally ill patients despite different work schedules and practice settings, particularly with junior physicians often rotating through different specialities and clinical settings.

Physicians and nurses were introduced to palliative care in medical and nursing school respectively. Whilst nearly 98% of physicians and 95% of nurses were aware of palliative care services in their hospital, close to 15% of nurses were uncertain as to what palliative care entailed (Table 2). Most physicians and nurses were also aware of hospices although some had different conceptualisations of their role. Approximately 86% of physicians and 82% of nurses saw hospice care as *“a place for palliation and optimising quality of life”*. Close to 10% of nurses saw hospices as being *“a place for cancer patients”.*

**Table 2:** Perceptions of palliative care

	Physicians (***n*** = 44)	Nurses (***n*** = 156)	***p***-value
How important do you think the role of palliative medicine is in the care of terminally ill patients?* Mean (+/−sd)	4.84 (0.43)	4.72 (0.48)	0.076
**How beneficial do you think each patient group will benefit from palliative care?**† **Mean (+/−sd)**			
Elderly Patients	4.11 (0.84)	4.16 (0.74)	0.910
Patients with Dementia	4.30 (0.79)	3.74 (0.98)	0.001
Patients with Cancer	4.95 (0.21)	4.65 (0.59)	0.001
Patients with Organ Failure	4.80 (0.46)	4.48 (0.66)	0.002
How important do you think each of the following indications are for palliative care referral?* Mean (+/−sd)			
Physical symptom control	4.7 (0.55)	4.6 (0.60)	0.160
Psychological/emotional support	4.4 (0.70)	4.5 (0.65)	0.612
Discharge/placement planning	4.0 (0.96)	4.4 (0.71)	0.016
**Which of the following do you feel is the biggest barrier in considering/recommending a referral to palliative care for your patient?﻿**‡ **n(%) selecting**			
Own uncertainty about what palliative care entails	3 (6.8)	22 (14.6)	
Difficulty in prognostication / Unpredictable course disease	26 (59.1)	66 (43.7)	
Patients’ fear of abandonment	5 (11.4)	30 (19.9)	0.037
Medical costs/ Financial concerns	1 (2.3)	17 (11.3)	
I have no difficulties or concerns	9 (20.5)	16 (10.6)	

* As measured on the following scale: 1 “not at all important”; 2 “not so important”; 3 “somewhat important”; 4 “important”; 5 “very important”

† As measured on the following scale: 1 “not at all beneficial”; 2 “not so beneficial”; 3 “somewhat beneficial”; 4 “beneficial”; 5 “very beneficial”

All p-values for * and † obtained from Wilcoxon Rank Sums test

All p-values for ‡ obtained from chi square test

### Indications for palliative care referral

Physicians and nurses acknowledged the import of palliative care amongst patients with terminal illnesses, with mean scores of 4.84 and 4.72 respectively on a 5-point Likert scale (Table 2). Both groups placed similar importance upon palliative care for physical symptom control, psychological and emotional support, and discharge and placement planning.

However, nurses tended to believe that elderly patients and patients with dementia were less likely to benefit from palliative care (3.74 vs 4.30, *p* = 0.001).

While majority of the physicians and nurses viewed difficulty prognosticating their patients’ disease progression as the biggest barrier to palliative care referrals (59.1 and 43.7% respectively), nearly 20% of nurses also believed that referral to palliative care could be construed by the patient as abandonment. About 11% of nurses also believed palliative care referrals could be financially costly.

### Self-reported behaviours

Only about 43% of nurses would discuss issues regarding the extent of care and care determinations with their patients and only 42% of nurses would always *“have conversations about palliative care to terminally ill patients”*. This contrasts starkly with physicians who reported that they would *“speak about the extent of care”* and care determinations with their patients and *“have conversations about palliative care to terminally ill patients”* (88.9% and 77.8% respectively, vs 42.9% and 41.7%, *p* = 0.001).

However, nurses were more likely than physicians to discuss palliative care options when patients are first diagnosed with a life limiting illness (73.7% vs 34.9%, p = 0.001). Nearly 11% of physicians would defer to the palliative care team to discuss these issues whilst 89% of physicians would include the family in these discussions.

Other disparities in introducing palliative care and approaches to end of life conversations between the participants are summarised in Table 3.

**Table 3:** Self-reported behaviours

	Physicians (***n*** = 44)	Nurses (***n*** = 156)	***p***-value
Always speak about extent of care to terminally ill patients* n(%) agree	40 (88.9)	67 (42.9)	0.001
Always have conversations about palliative care to terminally ill patients* n(%) agree	35 (77.8)	65 (41.7)	0.001
When would you consider a referral to palliative care?* n(%) agree			
When first diagnosed with life limiting illness	15 (34.9)	115 (73.7)	0.001
At symptom onset	43 (97.7)	128 (82.6)	0.011
When illness becomes terminal	44 (100.0)	150 (96.2)	0.187
If family or patient demonstrate distress	40 (93.0)	113 (72.4)	0.005
**How confident are you in explaining the role of palliative care to patients?**† **Mean (+/−sd)**	3.7 (0.74)	3.2 (0.86)	0.001
How do you first communicate the option of palliative care to patients and families?* n(%) selecting			
Hold a patient and/or family conference	39 (88.6)	57 (37.7)	
Refer to palliative care	5 (11.4)	87 (57.6)	0.001
Do not broach topic	0 (0.0)	7 (4.6)	
**How confusing do you find the different terminologies used (e.g terminal care, best supportive care, comfort care) are?**‡ **Mean (+/sd)**			
**How much do you agree or disagree with the following statements about palliative care?**§ **Mean (+/−sd)**	2.7 (1.06)	2.7 (0.98)	0.855
PC referrals are an underutilized service	3.6 (0.99)	3.4 (1.06)	0.184
Nurses should be included more in end of life conversations	4.3 (0.59)	4.0 (0.80)	0.015
There should be more PC training to healthcare workers	4.6 (0.50)	4.2 (0.72)	0.003
The general public does not know enough about PC	4.6 (0.54)	4.2 (0.82)	0.007

† As measured on the following scale: 1 “not at all confident”; 2 “not so confident”; 3 “somewhat confident”; 4 “confident”; 5 “very confident”

‡ As measured on the following scale: 1 “not at all confusing”; 2 “not so confusing”; 3 “somewhat confusing”; 4 “confusing”; 5 “very confusing”

§ As measured on the following scale: 1 “strongly disagree”; 2 “disagree”; 3 “neutral”; 4 “agree”; 5 “strongly agree”

All p-values for * obtained from chi square test

All p-values for †, ‡ and § obtained from Wilcoxon Rank Sums test

There was strong agreement that more stakeholders involved should be educated or trained in palliative care. High mean scores ranging from 4 to 4.6 were observed for statements such as *“Nurses should be included more in end of life conversations”, “There should be more palliative training to healthcare workers”* and *“The general public does not know enough about palliative care”.*

### Subgroup analysis

Subgroup analysis of our survey was performed comparing senior (consultants and registrars) versus junior (house and medical officers) physicians, and senior (nursing clinicians and managers) versus junior (enrolled and registered) nurses (Table 4). Senior physicians reported being more confident in explaining the role of palliative care to their patients as compared to junior physicians (3.95 vs 3.42, *p* = 0.020), but no statistical difference was  noted between senior and junior nurses. There was also no statistical difference between senior and junior physicians, and senior and junior nurses, with regards to initiating a referral upon first diagnosis of a life-limiting illness or at symptom onset.

**Table 4:** Seniors vs Juniors

	**Senior Physicians** **(*n*** **= 22)**	**Junior Physicians** **(*n*** **= 21)**	***p*-value**
When would you consider a referral to palliative care?* n(%) agree			
When first diagnosed with life limiting illness	6 (28.6)	8 (38.1)	0.513
At symptom onset	21 (95.5)	21 (100)	0.323
**How confident are you in explaining the role of palliative care to patients?† Mean (+/−sd)**	3.95 (0.6)	3.42 (0.8)	0.020
	**Senior Nurses** **(*n*** **= 45)**	**Junior Nurses** **(*n*** **= 111)**	***p*-value**
When would you consider a referral to palliative care?* n(%) agree			
When first diagnosed with life limiting illness	32 (71.1)	83 (74.8)	0.638
At symptom onset	34 (75.6)	94 (85.5)	0.140
**How confident are you in explaining the role of palliative care to patients?† Mean (+/−sd)**	3.07 (0.8)	3.24 (0.9)	0.273

† As measured on the following scale: 1 “not at all confident”; 2 “not so confident”; 3 “somewhat confident”; 4 “confident”; 5 “very confident”

All p-values for * obtained from chi square test

All p-values for †, ‡ and § obtained from Wilcoxon Rank Sums test

## Discussion

In answering its primary research question, this study revealed that a large majority of nurses and physicians at the Department of Internal Medicine at Singapore General Hospital recognise the importance of palliative care. However, effective utilisation of palliative care services continues to be undermined by gaps in knowledge, prognostication and communication. These gaps appear to stem from unfamiliarity with palliative care’s shift from a prognosis-based model to a needs-based model and failure to appreciate its merits in supporting patients with non-cancer diagnoses and chronic life-limiting conditions [27]. Indeed, this study reveals that misconceptions on the role of prognosis as an entry criteria inhibit up to 60% of physician initiated referrals to palliative care.

Scrutiny of the data captured in this survey also highlights different misconceptions between physicians and nurses about palliative care. Physicians tend to see palliative care services as being primarily for the palliation of physical symptoms in terminally ill cancer and non-cancer patients and do not seem to appreciate palliative care’s role in providing emotional and psychological support. Physicians also appear to be less likely to appreciate palliative care’s role in providing longitudinal support for patients and their families upon discharge as highlighted by lesser tendencies to refer appropriate cases to palliative care services.

Nurses in this study, on the other hand, appreciated palliative care’s holistic and longitudinal support even after hospital discharge. However, they were also more likely to voice concerns over their prognostication and communication skills and more likely to struggle when contending with sociocultural nuances. This may be reflected in the fact that up to 58% of nurses were unlikely to introduce or discuss palliative care services with patients and up to 60% who preferred to defer to the palliative care team to introduce themselves and their roles.

Underlining these lapses in understanding of palliative care and reaffirming the need for effective education of physicians and nurses on a national scale is the impact of regnant sociocultural considerations on local end of life practices. Ho et al. [28]‘s, Krishna [29]‘s, Yang et al. [30]‘s, Foo et al. [31]‘s, Chai et al. [32]‘s and Ching et al. [33]‘s studies which posit that prevailing sociocultural beliefs, particularly those attributed to Confucian based concepts of filial piety [34–41], may hamper the expansion of palliative and hospice care [29, 42–45] are particularly noteworthy here. These notions may shed further light on culturally-entrenched motivations behind delays to early and appropriate referrals.

There are three local interpretations of filial piety that have been especially influential to local end of life care [28, 46, 47]. One is the duty to ‘maintain hope’. Failure of the family and caregivers to do so is believed to precipitate depression and the hastening of death [48, 49]. This wish to ‘maintain hope’ has been seen to propagate local practice of collusion [43, 50, 51], resulting in families rejecting palliative and hospice care services to ‘protect’ the patient from ‘bad’ news. Such rejection is also attributed to another closely related local interpretation of filial piety – the duty of ‘non-abandonment’ [29]. As hospice care may suggest that a ‘cure’ is no longer possible, acceptance of a referral may be viewed as tantamount to abandonment of the patient and the loss of hope. Recommending such care may be a cause of concern for local healthcare professionals as highlighted by nearly 20% of the nurses and more than 10% of the physicians in this survey. This leads to the third local interpretation of filial piety which is the duty of ‘continued support’. This relates to family-led care and support of the patient. A family’s failure to meet this duty is fed in part by the mistaken belief that involvement of hospice and palliative care interventions would lead to reduced family involvement. This misconception is rooted in the notion that the ‘immediate’ role of the family would be diminished, at least with regards to the provision of the patient’s physical care. Acceptance of palliative and hospice care is thus regarded as unacceptable behaviour. These three interrelated [52] local interpretations of filial piety colours notions of palliative care [29, 42–45, 53] as does the oft-assumed concept of ‘hospice related care’ which paints hospices as merely ‘somewhere to go to die’ [54].

The combination of knowledge gaps, unfamiliarity with prognostication and the changing roles of palliative and hospice care may explain why few physicians in this survey would consider initiating a referral upon first diagnosis of a life-limiting illness. Rodriguez et al. [55]. suggest that such hesitancy may in part stem from the dated notion that palliative care consultations are ‘reserved’ for patients at the end of life. Our data further suggests that such misconceptions pervade all levels of training and experience, affirming the need for more stakeholder engagement in the form of a two-pronged approach – advancing knowledge and skills in palliative care and addressing sociocultural misconceptions.

In line with the World Health Organisation’s recommendation that requisite levels of competence in palliative care be rendered compulsory in basic medical professional qualifications [56], establishing common awareness and adherence to national strategies and guidelines such as those delineated by the Singapore Hospice Council should be considered in the curation and early stages of local palliative care education programs [57]. Returning to the concept of a needs-driven palliative care, training in palliative care amongst healthcare workers are imperative. These programs must include strategies to equip healthcare professionals with skills in prognostic communications [58, 59] and the use of validated needs assessment tools. Use of evidence based methods and care approaches will enhance confidence of healthcare professionals and help evolve local understanding. Standardised instruments such as the Integrated Palliative Care Outcome Scale (IPOS) and Palliative Care Needs Assessment Tool: Progressive Disease-Heart Failure (NAT: PD-HF) may help physicians and nurses identify patients who could benefit from palliative care involvement [60, 61].

As a result of survey findings and prevailing data of a wider ‘existential’ threat to wider, non-cancer related referrals for palliative care services evidenced in recent studies in Singapore, further training programs focusing on changing minds, enhancing cultural sensitivity and equipping healthcare professionals with requisite clinical and communication skills ought to be planned. In tandem, further engagement with the public is proposed to overcome socioculturally related misconceptions and to enhance understanding of palliative and hospice care’s role to all patients that need them.

### Study limitations

Although our study had a considerable 32.3% response rate, the low response rate of 22% among physicians may not be representative and constitutes a limitation of this study. As our survey was distributed to participants within the hospital’s internal medicine department, it may not be generalisable to other medical subspecialities or surgical specialties where the threshold for specialist palliative care referral may differ. Limited to physicians and nurses, our results do not represent all members of the multidisciplinary team which may also include social workers, therapists and pharmacists. As levels of palliative care training received by physicians and nurses were not ascertained, palliative-trained internists or nurses could have influenced perceptions and the study’s results.

## Conclusions

We believe this survey of physician and nurse perceptions of palliative care in internal medicine in Singapore General Hospital reflects a wider gap in understanding of palliative care in Singapore, given the presence of corroborative findings on gaps in communication and prognostication skills and a general misunderstanding of palliative care’s role amongst physicians and nurses. Given such wide ranging knowledge and skill gaps and misconceptions, we opine that there is a need for a concerted national education program to target these barriers to palliative care’s effective use as they likely persist across clinical settings. This program must focus on increasing communication skills and cultural sensitivity even as government led initiatives on educating the public to palliative care’s wider role continue. At the same time this data also lends weight to efforts by local medical and nursing schools to increase awareness of palliative care. We hope that these insights will be of use to our colleagues in Southeast Asia and beyond and we look forward to continuing this discussion.

## Data Availability

The datasets used and/or analysed during the current study are available from the corresponding author on reasonable request.

## Abbreviations

SGH: Singapore General Hospital
IPOS: Integrated Palliative Care Outcome Scale
NAT: PD-HF: Palliative Care Needs Assessment Tool: Progressive Disease-Heart Failure
COPD: Chronic obstructive pulmonary disease

## Footnotes

1 A five-point Likert scale was used (1 = not at all beneficial, 2 = not so beneficial, 3 = somewhat beneficial, 4 = beneficial, 5 = very beneficial).

2 A five-point Likert scale was used (1 = not at all important, 2 = not so important, 3 = somewhat important, 4 = important, 5 = very important).

3 A five-point Likert scale was used (1 = not at all confident, 2 = not so confident, 3 = somewhat confident, 4 = confident, 5 = very confident).

4 A five-point Likert scale was used (1 = not at all confusing, 2 = not so confusing, 3 = somewhat confusing, 4 = confusing, 5 = very confusing).

5 A five-point Likert scale was used 1 = strongly disagree, 2 = disagree, 3 = neutral, 4 = agree, 5 = strongly agree).
